# Bipolar disorder and cannabis abuse. A clinical case report

**DOI:** 10.1192/j.eurpsy.2023.1486

**Published:** 2023-07-19

**Authors:** S. Villa, R. Obrador, J. M. Crespo

**Affiliations:** Psychiatry, Bellvitge Universitary Hospital, L’Hospitalet de Llobregat, Spain

## Abstract

**Introduction:**

Cannabis or marijuana is a common substance of abuse. Its active compounds are Delta-9-tetrahydrocannabinol (Δ9-THC or THC), cannabidiol or nabiximol. The last two ones might have a therapeutic effect in some mental disorders. THC is a toxic substance that has euphoric, sedative and antalgic effects. It is the third most consumed psychoactive substance in the world, with 10% of people consuming it with an abusive patron.

The comorbidity of Bipolar Disorder (BD) and the cannabis abuse takes place in a 20% of the patients in some series. This has been related with a worse prognostic for the BD, being especially related to apparition of more episodes of mania.

We did a review of both disorders due to a case of a patient we had admitted to the psychiatry department of the Bellvitge Universitary Hospital with a debut of hippomania and history of cannabis consumption.

**Objectives:**

To expose a clinical case and to do a review of the literature related to BD and cannabis abuse.

**Methods:**

It is a one patient report of a 35 year old male that was a habitual consumer of cannabis. He achieved a consume of 1g per day. He began consuming it on December 2020, until 4-5 days before the hospitalisation on March 2022. His hospital admittance was due to a debut with hippomania clinical features.

Review of various scientific articles related to both disorders.

**Results:**

Our case clinical features were mainly an alteration in his conduct right after cannabis withdrawal. It consisted in mental hyper clarity, increased speed of his thought, insomnia, inadequacy, hyperactivity and increased energy; hipersexuality and wellness feeling.

His development was favourable with an olanzapine based treatment, later switched to aripiprazole. After the hospitalisation, his symptoms have been mainly related to the anxiety spectrum, due to a basal neurotic personality. He presented some depressive symptoms, but not with entity of decompensation. He hasn’t consumed cannabis since the admittance.

It’s been described that substance abuse is related to retardation on the diagnosis. Also, this comorbidity is related to a worse development in both disorders. In the case of BD, cannabis consumption has been related to more episodes of mania.

Lithium is the only treatment proved to improve both disorders at the same time.

Comorbidity for affective disorders with substance abuse has been described as a risk factor for suicide, overdose and homicide.

**Conclusions:**

Cannabis seems directly related with the onset and the exacerbation of a BD. This relation seems bilateral, since an untreated mania might result in a cannabis abuse disorder. Worse prognosis for BD might be because comorbidity with cannabis abuse is related with worse adherence to treatment and more decompensations. Also, the abuse of substances can provoke retardation in the diagnosis. By now, lithium seems to be the only treatment with proved efficacy treating comorbidity of both disorders.

**Disclosure of Interest:**

None Declared

